# Influence of a Prenatal Fruit and Vegetable Prescription Program on Diet and Household Food Security in a Low-Income, Urban Community

**DOI:** 10.1007/s10995-025-04212-5

**Published:** 2025-12-18

**Authors:** Amy Saxe-Custack, Jenny LaChance, Gayle Shipp, Diana Haggerty

**Affiliations:** Charles Stewart Mott Department of Public Health, Michigan State University-Hurley Children’s Hospital Pediatric Public Health Initiative, 200 E 1st St, Flint, MI 48502 USA

**Keywords:** Fruit and vegetable prescriptions, Diet, Pregnancy, Food security, Low-income

## Abstract

**Introduction:**

A prenatal fruit and vegetable prescription program (FVPP) was introduced in Flint, Michigan to increase access to fresh produce during pregnancy. This program provides $15 fresh fruit and vegetable prescriptions to all prenatal patients during office visits, redeemable at a local farmers market and mobile market/food hub. The current study assessed changes in diet and food security throughout pregnancy among patients exposed to the prenatal FVPP.

**Methods:**

This non-controlled longitudinal trial included prenatal patients at two low-income urban clinics presenting before 16 weeks’ gestation. Participants completed surveys to assess diet (one automated 24-hour dietary recall) and food security (US Household Food Security Module: Six Item Short Form - National Center for Health Statistics), with follow-up surveys at mid-pregnancy and postpartum.

**Results:**

A total of 118 pregnant women (mean age 26.32 ± 5.04 years, range 18–39 years) enrolled in the current study. Most identified as Black/African American (54.2%, *n* = 64), received Medicaid (66.9%, *n* = 79), and participated in The Special Supplemental Nutrition Program for Women, Infants, and Children or WIC (62.4% at midpoint). Participants received an average of 8.81 ± 2.30 fruit and vegetable prescriptions, with two-thirds redeeming at least one (66.9%, *n* = 79). Household food security worsened from baseline to midpoint (*p* = 0.076) but improved from midpoint to postpartum (*p* = 0.013). Participants reported notable changes in dietary behaviors at critical points during their pregnancy. Primarily, significant improvements in mean daily consumption of fruits and vegetables (*p* = 0.027); total vegetables (*p* = 0.015); and vegetables excluding potatoes (*p* = 0.030) were observed from early pregnancy to midpoint. Alternatively, mean daily intake of fruits and vegetables (*p* = 0.007) and total vegetables (*p* = 0.029) decreased between midpoint in pregnancy and the early postpartum period.

**Discussion:**

This study reveals the influence of a prenatal FVPP on diet and food security among prenatal patients living in one low-income, urban community. Results signal an urgent need for coordinated and comprehensive maternal supports that better address food and nutrition security during pregnancy and the early postpartum period.

## Significance

Fruit and vegetable prescription programs have demonstrated positive impacts on food security, diet, and markers of health among adults and youth; still, little is known about their effectiveness during pregnancy, a vital period during which maternal nutrition and lifestyle choices influence the health of mother and child. Findings of this study suggest that fruit and vegetable prescriptions distributed during pregnancy are associated with notable improvements in dietary intake during critical stages of pregnancy; however, modifications are necessary to better coordinate and tailor nutrition resources to enhance perinatal outcomes.

## Introduction

Pregnancy is a critical period during which dietary intake and lifestyle choices influence maternal and child health (Procter & Campbell, 2014; Prado & Dewey, 2014). Proper nutritional status during this time supports optimal fetal growth and brain development and prevents adverse pregnancy outcomes, infant mortality, birth defects, and chronic disease (Shapira, 2008). Unfortunately, there are widespread barriers to accessing high-nutrient foods in many low-income communities, including limited grocery stores, poor quality fresh foods, inadequate transportation options, and financial constraints (Saxe-Custack et al., 2018; Saxe-Custack et al., 2019c; Mayfield et al., 2017; Phelan et al., 2021). Moreover, many women have multiple competing priorities which make operationalizing dietary recommendations during pregnancy difficult (Grenier et al., 2021).

To address existing challenges, Food as Medicine interventions are emerging in various forms, including medically tailored meals, grocery provisions, and produce prescriptions. Initiatives focused on pregnant individuals generally aim to improve food security and dietary intake while mitigating adverse maternal and infant health outcomes (Balis et al., 2023). Still, Food as Medicine interventions during pregnancy are relatively new, and the existing literature to support or contradict their effectiveness is limited (Merchant et al., 2023). For example, these interventions have shown promise in improving food security during pregnancy; while findings related to dietary intake, particularly consumption of fruits and vegetables, have yielded mixed results (Merchant et al., 2023). Food as Medicine initiatives have also demonstrated small, positive impacts on health outcomes, but more research is needed to better characterize program components and assess their effectiveness.

Fruit and vegetable prescriptions, one type of Food as Medicine intervention, have grown and expanded over the last decade, becoming more widely available across the United States (US). Although differing in structure and design, the majority of fruit and vegetable prescription programs include “prescriptions” for fruits and vegetables issued by a healthcare provider to exchange at local farmers’ markets and food retailers. The positive impacts of these programs on food security, diet, and markers of health among adults (Bryce et al., 2017; Bhat et al., 2021) and youth (Saxe-Custack et al., 2021; Saxe-Custack et al., 2019a; Ridberg et al., 2019) have been documented. However, limited research exists on the impact of fruit and vegetable prescriptions specifically designed for pregnant individuals, particularly regarding their effects on diet and food security.

In 2016, the first fruit and vegetable prescription program (FVPP) exclusively for pediatric patients was introduced in Flint, Michigan. This program provides $15 fresh fruit and vegetable prescriptions to every patient (birth to 19 years) during office visits and is available at three large pediatric clinics in Flint. Exposure to this program has been associated with improvements in dietary patterns of young patients as well as household food security (Saxe-Custack et al., 2021; Saxe-Custack et al., 2019a; Saxe-Custack et al., 2020). Moreover, qualitative findings suggest that this FVPP serves as a vital resource when food dollars are limited (Saxe-Custack et al., 2018). In 2022, as an intentional effort to address barriers to accessing fresh fruits and vegetables during pregnancy, the FVPP that has served youth in Flint since 2016 was expanded to include prenatal patients (Saxe-Custack et al., 2024b). Study objectives are to assess changes in dietary patterns and food security throughout pregnancy among prenatal patients exposed to this FVPP.

## Methods

### Fruit and Vegetable Prescription Program (FVPP)

Currently available to all prenatal patients at two obstetrics and gynecology (OB/GYN) clinics, the FVPP provides one $15 prescription for fresh fruits and vegetables at each prenatal visit (maximum of 14 prescriptions). The prescriptions also serve as a reminder for health care providers to discuss the importance of healthy eating and food choices during pregnancy. Prescriptions may be redeemed at the downtown Flint Farmers' Market or Flint Fresh (a mobile market and food hub that delivers fresh produce boxes locally). The farmers’ market is open year-round on Tuesdays, Thursdays, and Saturdays from 9 AM until 5 PM. Flint Fresh (www.flintfresh.com) is open year-round and offers participant-selected fresh produce boxes that are delivered free of charge. Vendors treat the prescriptions ($15 each) as gift certificates or vouchers that may only be redeemed for fresh fruits and vegetables. Participants must use the entirety of their prescription in one transaction; multiple $15 prescriptions may be redeemed at one visit; and no change is provided. In partnership with Flint Farmers' Market and Flint Fresh, our research team has tracked and recorded prescription redemption rates since the program was initiated.

### Study Participants and Design

**Fig. 1:**
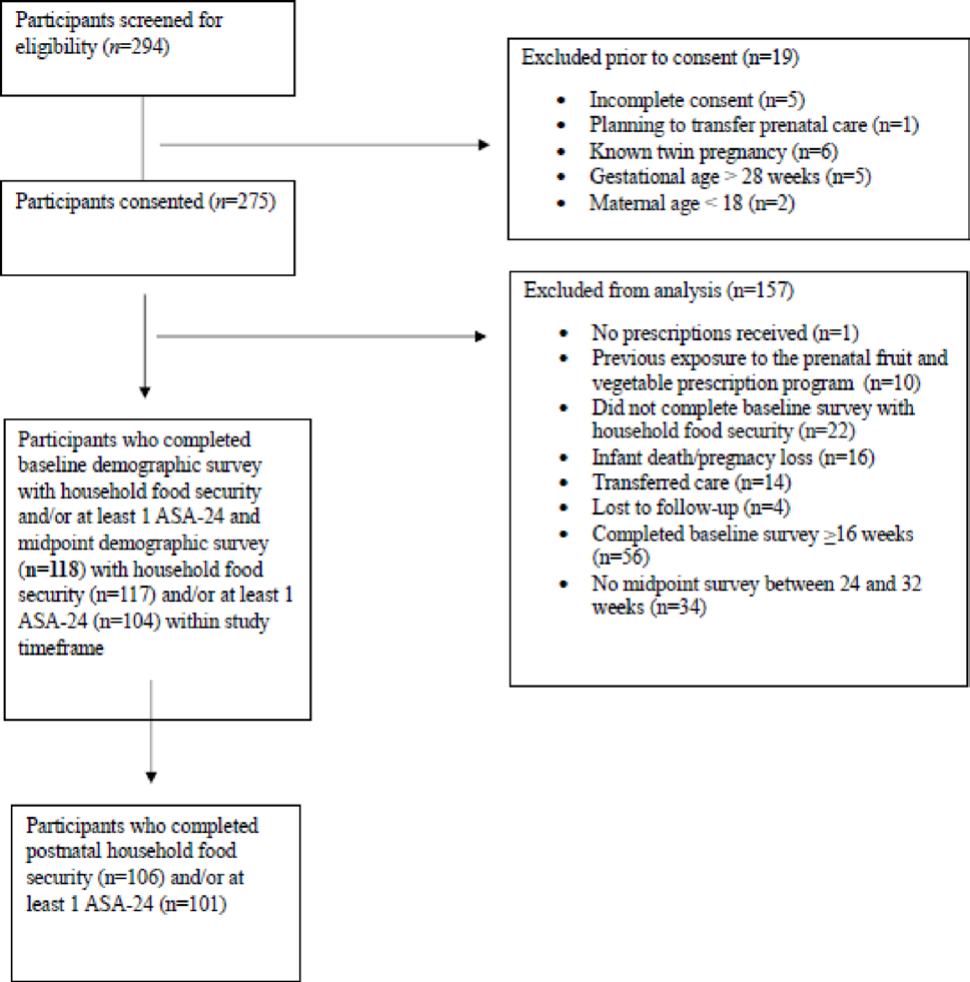
Study enrollment schematic showing the number of participants screened, consented, and included in baseline, midpoint, and postnatal analysis

A consecutive sample of patients who presented for their first prenatal appointment at two partnering OB/GYN clinics that offered the FVPP were approached for participation in a study to examine feasibility and preliminary effectiveness of the prenatal FVPP. Patients were eligible to participate if they were presenting for their first appointment; were between the ages of 18 and 43 years of age; spoke English; and provided written informed consent. Although all pregnant women received fruit and vegetable prescriptions, the current investigation excluded women with multiple or complicated pregnancies, or those with severe pre-existing illnesses, that may influence dietary behaviors and limit generalizability of study findings. Following consent and enrollment at partnering clinics, participants completed a series of demographic as well as survey questions to assess dietary patterns and food security. Baseline surveys were completed in-person at partnering clinics. Surveys were completed again at midpoint of pregnancy and postpartum using a virtual platform. Data were collected from June 2022 through February 2024. As shown in Fig. 1, the current study includes a subsample of participants without previous exposure to the prenatal FVPP who enrolled and provided baseline data at < 16 weeks gestation. Midpoint surveys were included for participants who met inclusion criteria at baseline and completed household food security and/or at least one 24-hour dietary recall between 24- and 32-weeks gestation. Postpartum surveys were included for participants who met inclusion criteria at baseline and completed household food security and/or at least one 24-hour dietary recall at the final assessment. Demographic and survey data were collected electronically using the secure digital platform, Research Electronic Data Capture (REDCap). This study was conducted in accordance with the Declaration of Helsinki and approved by Michigan State University Institutional Review Board (Study 00006239 title "Prenatal Nutrition Prescription Program") (Figure 1).

### Dietary Assessment

Participants completed one 24-hour dietary recall at each timepoint (baseline, midpoint, and postpartum). Dietary data were recorded via the Automated Self-Administered 24-Hour (ASA-24) Dietary Assessment Tool developed by the National Cancer Institute (Subar et al., 2012). The ASA-24 respondent website guided participants through the completion of each dietary recall with automatic prompts to record details of each eating event, including food brands and portion sizes. Trained research staff explained the ASA-24 interface to participants and assisted those who experienced any difficulty.

The ASA-24 researcher website was then accessed to obtain participant food group and nutrient data files. The ASA-24 provides approximate values for intake of macronutrients and micronutrients based on the Food and Nutrient Database for Dietary Studies and food categories based on the United States Department of Agriculture’s (USDA) Food Patterns Equivalents Database.

### Household Food Security Assessment

Participants completed the US Household Food Security Module: Six Item Short Form (National Center for Health Statistics) to measure household food insecurity and hunger (Blumberg et al., 1999). The modules were completed at baseline, midpoint, and postpartum. The sum of affirmative responses to six questions served as the household’s raw score. Food security status may be interpreted based on a calculated raw score (0–1 = high/marginal food security; 2–4 = low food security; 5–6 very low food security). For the current analysis, a dichotomous summary score was also created (0–1 = high/marginal food security; 2–6 = low/very low food security).

### Sociodemographic Characteristics

Participants self-reported age, race, education, medical insurance provider, participation in The Special Supplemental Nutrition Program for Women, Infants, and Children (WIC), and participation in The Supplemental Nutrition Assistance Program (SNAP). Gestational age was calculated based on infants’ estimated delivery date.

### Sample Size Calculation

To ensure there was an adequate sample size for the primary outcomes (change of 0.25 ± 0.80 cups of fruits and vegetables), at least 110 participants with pre and post matched data pairs were required for a power of 0.90 with an alpha of 0.05.

### Statistical Analyses

Participant characteristics and key outcomes are reported for those who met inclusion criteria for the current study. Descriptive analysis also examined the number of prescriptions redeemed. Changes in categorical data were examined using McNemar chi-square tests, and changes in mean values were analyzed using the paired t-test. Additionally, analyses of key outcomes were completed for those who redeemed at least one prescription and for those who redeemed no prescriptions. Analyses were performed in IBM SPSS Statistics version 27. Statistical significance was two-sided and defined at alpha level 0.05.

## Results

### Participant Characteristics

A total of 118 pregnant women enrolled in the study and provided baseline data. Mean age at enrollment was 26.32 ± 5.04 years (range of 18–39 years), and the average gestational age at baseline was 10.92 ± 2.76 weeks (range of 5–15 weeks). Noting that race categories were not mutually exclusive, the majority of study participants (54.2%, *n* = 64) reported race as African American; 43.2% (*n* = 51) reported race as White; and 5.1% (*n* = 6) selected other or did not respond. Most participants reported having a high school diploma or less (53.4%, *n* = 63), were receiving Medicaid (66.9%, *n* = 79), and were enrolled in WIC at the midpoint assessment (62.4%, *n* = 73). Participant characteristics are provided in Table 1. Participants received an average of 8.81 ± 2.30 (range of 2–14) fruit and vegetable prescriptions from their first prenatal visit until their final postpartum visit. At baseline, there were no significant differences in key dietary measures (total fruit and vegetables, total fruits, whole fruits, total vegetables, and vegetables excluding while potatoes) between those who redeemed prescriptions and those who did not redeem prescriptions.

**Table 1:** Baseline characteristics and program exposure

Participant characteristics		Mean ± SD (*n* = 118)
Age (years)		26.32 ± 5.04
Gestational age at baseline (weeks)		10.92 ± 2.76

Participant characteristics		Frequency (*n* = 118)
Race (*not mutually exclusive categories*)	African American	54.2% (*n* = 64)
	White	43.2% (*n* = 51)
	Other/Not reported	5.1% (*n* = 6)
Education	High school degree or less	53.4% (*n* = 63)
	Some college/Technical school/Associate’s degree	31.4% (*n* = 37)
	Bachelor’s degree	8.5% (*n* = 10)
	Graduate degree	5.1% (*n* = 6)
	Other/Prefer not to answer	1.7% (*n* = 2)
Insurance (*not mutually exclusive categories*)	Medicaid	66.9% (*n* = 79)
	Private	24.6% (*n* = 29)
Participation in the Special Supplemental Nutrition Program for Women, Infants, and Children (WIC)	Yes at baseline Yes at midpoint Yes at postnatal	42.4% (*n* = 50) 62.4% (*n* = 73) 71.0% (*n* = 76)
Participation in the Supplemental Nutrition Assistance Program (SNAP)	Yes at baseline Yes at midpoint Yes at postnatal	48.6% (*n* = 55) 48.7% (*n* = 57) 59.8% (*n* = 64)

Program exposure		Mean ± SD and frequency (*n* = 118)
Number of prescriptions received (baseline to postpartum)		8.81 ± 2.30
Redeemed at least 1 prescription	Yes	66.9% (*n* = 79)
	No	33.1% (*n* = 39)

Participants in the current study redeemed between 0 and 13 prescriptions during the study period, and there was a low-moderate correlation (*r* = 0.36, *p* < 0.001) between the number of prescriptions received and redeemed. Approximately one third of participants (33.1%, *n* = 39) redeemed no prescriptions; 27.9% (*n* = 33) redeemed 1 or 2 prescriptions; 17.0% (*n* = 20) redeemed 3 or 4 prescriptions; and 22.0% (*n* = 26) redeemed 5 or more prescriptions. For those who redeemed prescriptions, the redemption mode was 1 prescription, and the mean was 3.82 ± 3.02 prescriptions. Although sample size was insufficient to assess impact of redemption based on number of prescriptions redeemed, Tables 2 and 3 present selected analyses of key outcomes for the entire sample as well as for those who redeemed one or more prescriptions versus those who redeemed no prescriptions.

**Table 2:** Dietary outcomes by redemption status

	Sample size	Baseline	Midpoint	*P* value	Sample size	Midpoint	Postnatal	*P* value
*Total fruit and vegetables (cup eq.)*								
Overall	*n* = 104	2.23 ± 1.68	3.01 ± 3.20	0.027	*n* = 101	2.82 ± 2.47	2.13 ± 1.86	0.007
Redeemed	*n* = 72	2.29 ± 1.68	3.21 ± 3.53	0.050	*n* = 71	2.91 ± 2.53	2.21 ± 1.93	0.031
Not redeemed	*n* = 32	2.12 ± 1.68	2.56 ± 2.28	0.309	*n* = 30	2.59 ± 2.35	1.93 ± 1.69	0.089
*Fruits (cup eq.)*								
Overall	*n* = 104	1.04 ± 1.32	1.36 ± 2.59	0.241	*n* = 101	1.18 ± 1.61	0.92 ± 1.29	0.095
Redeemed	*n* = 72	1.08 ± 1.30	1.59 ± 3.03	0.182	*n* = 71	1.31 ± 1.81	0.86 ± 1.26	0.041
Not redeemed	*n* = 32	0.95 ± 1.38	0.86 ± 0.93	0.782	*n* = 30	0.90 ± 0.95	1.00 ± 1.38	0.672
*Whole fruit (cup eq.)*								
Overall	*n* = 104	0.65 ± 1.08	0.77 ± 2.32	0.631	*n* = 101	0.59 ± 1.18	0.45 ± 0.93	0.316
Redeemed	*n* = 72	0.68 ± 1.10	0.91 ± 2.75	0.503	*n* = 71	0.64 ± 1.34	0.39 ± 0.78	0.141
Not redeemed	*n* = 32	0.59 ± 1.06	0.46 ± 0.65	0.568	*n* = 30	0.46 ± 0.67	0.60 ± 1.22	0.470
*Total vegetables (cup eq.)*								
Overall	*n* = 104	1.19 ± 0.98	1.65 ± 1.68	0.015	*n* = 101	1.63 ± 1.69	1.23 ± 1.20	0.029
Redeemed	*n* = 72	1.20 ± 1.01	1.62 ± 1.56	0.066	*n* = 71	1.61 ± 1.55	1.35 ± 1.30	0.218
Not redeemed	*n* = 32	1.17 ± 0.91	1.70 ± 1.96	0.101	*n* = 30	1.69 ± 2.01	0.93 ± 0.90	0.050
*Vegetables excluding white potatoes (cup eq.)*								
Overall	*n* = 104	0.70 ± 0.76	0.96 ± 0.96	0.030	*n* = 101	0.95 ± 0.93	0.78 ± 0.98	0.200
Redeemed	*n* = 72	0.69 ± 0.77	0.95 ± 0.99	0.077	*n* = 71	0.95 ± 0.95	0.88 ± 1.05	0.664
Not redeemed	*n* = 32	0.74 ± 0.75	0.98 ± 0.90	0.207	*n* = 30	0.95 ± 0.91	0.56 ± 0.77	0.065

**Table 3:** Household food security during pregnancy and postpartum

Food insecurity^a^	Baseline to midpoint (*n* = 117)			Midpoint to postpartum (*n* = 106)		
	Baseline Percentage food insecure	Midpoint Percentage food insecure	P-value	Midpoint Percentage food insecure	Postnatal Percentage food insecure	P-value
Mean ± Standard Deviation	1.10 ± 1.82	1.48 ± 2.02	0.017	1.54 ± 2.09	1.11 ± 1.89	0.002
Overall Food Insecure	26.5%	35.0%	0.076	34.9%	24.5%	0.013
Redeemed (*n* = 78)	26.9%	37.2%	0.096	35.6%	24.7%	0.057
Not redeemed (*n* = 39)	25.6%	30.8%	0.727	33.3%	24.2%	0.250

Screener questions^b^	Baseline to midpoint (*n* = 117)			Midpoint to postpartum (*n* = 106)		
	Baselinepercentage	Midpoint percentage	P-value	Midpointpercentage	Postnatalpercentage	P-value
1. The food that (I/we) bought just didn’t last, and (I/we) didn’t have the money to get more.	28.2%	42.7%	0.003	42.5%	30.2%	0.011
2. (We) couldn’t afford to eat balanced meals.	31.6%	35.9%	0.424	36.8%	21.7%	< 0.001
3. In the last 12 months, did (you/you or other adults in your household) ever cut the size of your meals or skip meals because there wasn’t enough money for food?	14.5%	21.4%	0.096	23.6%	17.9%	0.109
4. In the last 12 months, did you ever eat less than you felt you should because there wasn’t enough money for food?	16.2%	18.8%	0.629	20.8%	16.0%	0.332
5. In the last 12 months, were you ever hungry but didn’t eat because there wasn’t enough money for food?	9.4%	13.7%	0.332	13.2%	12.3%	1.000

^a^Assessed with U.S. Household Food Security Survey Module: Six-Item Short Form (Blumberg et al., 1999)(possible scores 0–6; lower = greater food security) with scores of 0–1 coded as food secure and scores of 2–6 coded as food insecure

^b^Responses of “Often true” or “Sometimes true” on questions 1 and 2, and “Yes” on 3, 4, and 5 are coded as affirmative (yes = 1). There is a follow-up question (3a) to 3 that asks about frequency that is included in the score but is not shown here. For 3a, responses of “Almost every month” and “Some months but not every month” are coded as affirmative (yes = 1). The sum of affirmative responses to the six questions is the household’s raw score on the scale

### Dietary Intake

As shown in Table 2, participants reported significant improvements in mean daily consumption of key dietary components from baseline to midpoint. Mean daily intake of total fruits and vegetables increased (*p* = 0.027) from baseline (2.23 ± 1.68 cups) to midpoint (3.01 ± 3.20 cups) among study participants. Importantly, mean daily intake of total vegetables increased (*p* = 0.015) from baseline (1.19 ± 0.98 cups) to midpoint (1.65 ± 1.68 cups) among study participants. Mean daily consumption of vegetables excluding potatoes also improved (*p* = 0.030) from baseline (0.70 ± 0.76 cups) to midpoint (0.96 ± 0.96 cups) among study participants. When assessing change in fruit and vegetable intake based on prescription redemption, participants who redeemed at least one prescription reported an increase in mean daily consumption of total fruits and vegetables (*p* = 0.050) from baseline (2.29 ± 1.68 cups) to midpoint (3.21 ± 3.53 cups). Change in mean daily intake of total fruits and vegetables among study participants who did not redeem prescriptions was not significant (*p* = 0.309) from baseline (2.12 ± 1.68 cups) to midpoint (2.56 ± 2.28 cups).

As shown in Table 2, consumption of fruits and vegetables seemed to worsen from midpoint to the early postpartum period. Mean daily intake of total fruits and vegetables decreased (*p* = 0.007) from midpoint (2.82 ± 2.47 cups) to the postpartum assessment (2.13 ± 1.86 cups). Mean daily consumption of total vegetables similarly decreased (*p* = 0.029) from midpoint (1.63 ± 1.69 cups) to postpartum (1.23 ± 1.20 cups) among study participants. When assessing change in fruit and vegetable intake based on engagement in the FVPP, participants who redeemed at least one prescription experienced a significant decrease (*p* = 0.031) in mean daily consumption of total fruits and vegetables from midpoint (2.91 ± 2.53 cups) to postpartum (2.21 ± 1.93 cups) as well as a significant decrease (*p* = 0.041) in mean daily intake of total fruits from midpoint (1.31 ± 1.81 cups) to postpartum (0.86 ± 1.26 cups). Alternatively, those who did not redeem prescriptions experienced a notable decrease (*p* = 0.050) in mean daily consumption of total vegetables from midpoint (1.69 ± 2.01 cups) to postpartum (0.93 ± 0.90 cups) as well as a decrease (*p* = 0.065) in mean daily intake of vegetables excluding potatoes from midpoint (0.95 ± 0.91 cups) to postpartum (0.56 ± 0.77 cups).

### Household Food Security

As shown in Table 3, household food security worsened (*p* = 0.076) from baseline (food insecure = 26.5%) to midpoint (food insecure = 35.0%), suggesting that participants moved closer to food insecurity as pregnancy progressed. Moreover, a significantly higher proportion of participants (*p* = 0.003) responded affirmatively to the statement, “The food that (I/we) bought just didn’t last, and (I/we) didn’t have the money to get more” at midpoint (42.7%) when compared to baseline (28.2%). Household food security improved (*p* = 0.013) from midpoint (food insecure = 34.9%) to postpartum (food insecure = 24.5%), suggesting that participants felt more food secure in the weeks that followed childbirth when compared to mid-pregnancy. Additionally, a significantly lower proportion of participants (*p* = 0.011) responded affirmatively to the statement, “The food that (I/we) bought just didn’t last, and (I/we) didn’t have the money to get more” at postpartum (30.2%) when compared to midpoint (42.5%). Further, a lower proportion of participants (*p* < 0.001) responded affirmatively to the statement, “(We) couldn’t afford to eat balanced meals” at postpartum (21.7%) when compared to midpoint (36.8%). When assessing change in food security by prescription redemption status, there was a similar worsening of food security from baseline to midpoint regardless of engagement in the FVPP. However, participants who redeemed at least one prescription experienced a notable improvement (*p* = 0.057) in food security from midpoint (food insecure = 35.6%) to postpartum (food insecure = 24.7%).

## Discussion

Although most participants failed to meet dietary recommendations, noteworthy improvements in key dietary factors were observed between baseline (< 16 weeks gestation) and midpoint of pregnancy (24–32 weeks gestation), particularly among those who redeemed prescriptions. The potential impacts of the FVPP on dietary behaviors of pregnant women are consistent with literature that has shown a positive association between exposure to produce prescription programs and nutrition and health of participants (Saxe-Custack et al., 2021; Saxe-Custack et al., 2019a; Hager et al., 2023). Because women frequently alter dietary habits during pregnancy to promote healthy birth outcomes (Forbes et al., 2018), the current FVPP likely served as a tangible tool to support dietary improvements during this critical period of development.

Unfortunately, dietary behaviors that improved during pregnancy seemed to worsen in the weeks following childbirth. Previous research has indicated that participants in the FVPP are willing to explore new fruits and vegetables when purchasing with prescriptions (Saxe-Custack et al., 2022). However, when prescriptions are no longer available, participants return to previous shopping patterns to ensure household members will consume the foods purchased. Findings suggest that although this FVPP may have served as a support for women during their pregnancy, it was not sufficient to sustain dietary changes during the early postpartum period (when the prenatal FVPP ended). Additional related research has identified barriers to FVPP engagement, including transportation challenges, lost or expired prescriptions, and a desire for grocery store participation (Saxe-Custack et al., 2024a). Recent programmatic changes, including expansion to grocery stores as well as digitization of prescriptions, are designed to address these challenges. Still, results suggest that there is an enduring need to offer coordinated and consistent maternal nutrition supports throughout pregnancy with continued follow-up during the early postpartum period.

Unlike maternal dietary components, household food security worsened from baseline to midpoint, then improved appreciably from midpoint of pregnancy to the early postpartum period. Approximately 32% of women revealed at baseline that they “couldn’t afford to eat balanced meals”. At the postpartum assessment, that percentage had dropped to 22%; indicating that most study participants could afford to consume a healthy diet following the birth of their infants. Conversely, at baseline approximately 28% of participants indicated that “the food they bought just didn’t last, and we didn’t have the money to get more”. This percentage rose to 43% at midpoint and decreased to 30% at the postpartum visit. These results signal a notable worsening of food security and hunger during critical stages of pregnancy that subsequently improved after childbirth. Because food insecurity during pregnancy is associated with an increased risk of pregnancy complications, including higher gestational weight gain, gestational diabetes and pre-term birth (Laraia et al., 2010; Nguyen et al., 2024), addressing barriers to engagement in the FVPP is critically important. Future studies should examine engagement in the FVPP alongside utilization of other food and nutrition assistance programs, such as WIC and SNAP, to better coordinate and tailor resources to meet individual needs of participants.

Previous research related to dietary patterns during the prenatal period has shown that social determinants of health, including household income, influence diet (Aubert et al., 2022). Factors such as age, income, education, physical activity, and pre-pregnancy body mass index (BMI) have also shown associations to diet quality during pregnancy; however, there is a growing need to establish causal relationships (Aubert et al., 2022; Bodnar et al., 2002). Participation in nutrition assistance programs, such as WIC, has demonstrated improvements in diet quality among participants (Bodnar et al., 2002). However, many low-income pregnant women enrolled in WIC still fail to meet dietary recommendations, particularly for vegetables, whole grains, and micronutrients (Rojhani et al., 2021; Fowles & Gabrielson, 2005). Moreover, research has indicated that diet quality of many pregnant women who are enrolled in WIC requires improvement, particularly with regard to consumption of vegetables (Rojhani et al., 2021). The sample size in the current study was insufficient to explore synergistic effects of the FVPP with nutrition assistance programs, such as WIC. Moreover, data regarding shopping behaviors or benefit-specific usage was unavailable. It cannot be ruled out that the gradual increase in enrollment in WIC and SNAP programs from early pregnancy through the postpartum period was partially responsible for the increase in total fruit and vegetable consumption observed from early to mid-pregnancy in both groups. Unfortunately, we are unable to determine the extent to which changes in consumption patterns were attributable to the combined effect of one or more nutrition programs.

Findings related to dietary changes and food security suggest that the current prenatal FVPP should be modified to provide coordinated and consistent nutrition support to expectant women that carries into the early postpartum period. Dietary changes improved early in pregnancy, with pronounced improvements among those who redeemed at least one prescription. However, participants seemed to return to baseline intake of key dietary components following the delivery of their infants. These findings underscore the growing need to better understand how pregnant women engage with food assistance programs—such as WIC, SNAP, and FVPP—both individually and collectively, including how resources may be distributed within the household (e.g., shared with children). Although our study did not model the combined effects of these programs; dietary behaviors among participants revealed notable fluctuations, raising questions about whether current resources sufficiently meet their nutritional needs. Gaining a clearer understanding of the individual and combined impacts of these programs on nutrition and food security across different stages of pregnancy is critical for informing effective interventions and educational efforts. Future research should further examine the relationship between participation in prenatal FVPPs and dietary changes over time, ideally with a larger sample of pregnant women.

Limitations of the current study should be noted. The sample was small and limited to one geographic area. As such, results may not be generalizable to pregnant women living in dissimilar areas. However, it is important to note the community studied is not unlike numerous urban areas throughout the US that are severely lacking in resources and nutritional options. Pregnant women in Flint, like their children, too often face the double-burden of insufficient intake of nutrient-dense foods coupled with high intake of poor-quality, calorie-dense foods (Saxe-Custack et al., 2019b), and these challenges are prevalent throughout many low-income communities in the US (Brunst et al., 2013). As such, these findings are critical to understanding the unique needs of women living in communities similar to Flint. Although the dietary assessment tool used in the current study is robust, it is not without limitations in usability (Kupis et al., 2019). Additionally, the use of one 24-hour dietary recall may not be reflective of usual dietary patterns. Furthermore, many women completed only one follow-up survey and dietary assessment (midpoint or postnatal). Next, when analyzing food security data, the McNemar’s chi square test did not have sufficient power to detect a statistically significant difference between timepoints. As a result, the continuous score paired t-test was used to demonstrate the pattern persists and should be explored in future studies. Finally, current methods related to tracking of prescriptions fail to provide an exact date of prescription redemption. While nearly 70% of participants redeemed at least one fruit and vegetable prescription, information was unavailable regarding precise timing of redemption, shopping behaviors, or benefit-specific usage.

## Conclusion

This study provides important information regarding the influence of a prenatal FVPP on dietary patterns and food security among a sample of patients living in one low-income, urban community. Findings of this study suggest that the current FVPP is associated with improvements in dietary intake during critical stages of pregnancy; however, modifications are necessary to better address maternal diet and food security. Importantly, the current FVPP might be combined with federally funded food and nutrition assistance programs, such as WIC or SNAP, to better coordinate and adapt resources to meet individual needs of participants in an effort to enhance perinatal outcomes.

## Data Availability

Deidentified data supporting the findings of this study are available from the corresponding author, ASC, upon reasonable request.
